# Implementing patient- and family-centered rounds training with direct-observation assessment for medical students in the United States: a prospective feasibility study

**DOI:** 10.3352/jeehp.2026.23.18

**Published:** 2026-06-18

**Authors:** Emily Xiao, Erin Chen, Amit Pahwa

**Affiliations:** 1 Department of Pediatrics, Johns Hopkins School of Medicine, Baltimore, MD, USA; 2 Department of Pediatrics, Baylor College of Medicine, Houston, TX, USA; 3 Department of Pediatrics, Children’s Hospital of Philadelphia, Philadelphia, PA, USA; 4 Division of Pediatric Hospital Medicine, Johns Hopkins Hospital, Baltimore, MD, USA; Hallym University, Korea

**Keywords:** Undergraduate medical education, Teaching rounds, Clinical clerkship, Educational measurement, Health communication, United States

## Abstract

**Purpose:**

This study evaluated the implementation of a patient- and family-centered rounds (PFCR) educational intervention and a standardized assessment form developed to assess changes in medical students’ PFCR performance over time.

**Methods:**

From October 2023 to October 2024, medical students at Johns Hopkins School of Medicine attended a 1-hour PFCR simulation workshop during the Core Clerkship in Pediatrics. Students rotating at the main campus were assessed during rounds with a formative standardized form; all students received summative oral presentation and family rapport scores regardless of site. Performance was compared between students rotating in the first half (H1) and second half (H2) of the clerkship using Wilcoxon rank-sum tests with Holm correction for multiple comparisons. Linear mixed-effects models with student-level random intercepts were used to estimate changes across serial assessments.

**Results:**

Among the 74 students rotating at the main campus, assessment forms were completed for 61% of students, with a median of 3 completed forms per student. H1 and H2 students had similar scores on both the formative assessment form and summative evaluations. Both groups improved significantly in inviting patient and family concerns across serial assessments. Summative scores did not differ between students evaluated before and after the educational intervention or between students at the main campus and those at community hospitals.

**Conclusion:**

A structured PFCR educational session paired with a standardized assessment form was feasible to implement in a pediatric clerkship. Observed student-level median scores were at or above “meets expectations” across 6 PFCR domains, and no significant performance decline was observed among students rotating later in the clerkship. Further controlled studies are needed to determine whether this intervention improves PFCR skills compared with usual training.

## Graphical abstract

[Fig f1-jeehp-23-18]

## Introduction

### Background

The Institute for Patient- and Family-Centered Care and the American Academy of Pediatrics have recommended patient- and family-centered rounds (PFCR) in pediatric hospitals since 2012 [1]. Several frameworks have been developed to support PFCR implementation, including I-PASS, a mnemonic framework for structured handoff communication that was later adapted for rounds [2]. Use of this framework during PFCR has been associated with greater family and nurse engagement without prolonging rounds [3].

Simulation and other curricular approaches have been used to improve pediatric residents’ communication during PFCR [4]. In one implementation of an I-PASS workshop for residents, most participants agreed that they could engage families and interprofessional team members in developing a shared mental model during PFCR [5].

Medical students are also expected to present during rounds. In a national survey of pediatric clerkship directors in the United States, 85% of responding programs reported student participation in PFCR; however, the content, structure, and formality of preparatory sessions varied widely [6]. Workshops have been developed to prepare students for PFCR. For example, a workshop that combined video-based learning with small-group practice improved students’ comfort with presentations during PFCR [7]. Another workshop on “presenter empowerment actions,” based on behaviors of excellent presenters, increased interns’ confidence and intent to use these skills; medical students participated, but their outcomes were not reported [8]. Despite this work on self-reported comfort and confidence, students’ directly observed PFCR performance has received less attention.

### Objectives

The theoretical basis for this intervention was that deliberate practice through simulation, combined with direct observation and structured feedback during actual rounds, could build and reinforce communication skills specific to PFCR. At our institution, Johns Hopkins School of Medicine, we developed an educational session in which students simulated PFCR, and we then asked attendings to assess students’ performance during actual PFCR. We also developed a brief standardized assessment form based on a structured communication model and on skills expected at the medical student level (Supplement 1). We aimed to evaluate the feasibility of implementing this paired session and assessment form within the Core Clerkship in Pediatrics, as indicated by completion rates, the number of forms completed per student, and the proportion of domains that could be observed during actual rounds. With regard to effectiveness, we hypothesized that the session would improve PFCR performance as observed by pediatric faculty. We also hypothesized that end-of-clerkship oral presentation and family rapport scores would be higher among students at our main campus, where the assessment form was used, than among students at community hospital sites, where the form was not used, and would be higher after the intervention than during the pre-intervention period.

## Methods

### Ethics statement

The study was approved by the Johns Hopkins Medicine Institutional Review Board (#IRB00408754). Because the research involved no more than minimal risk, a waiver of documentation of consent was granted, and consent was obtained verbally. Students were given the opportunity to decline inclusion of their data in the study.

### Study design

This was a prospective, single-arm, quasi-experimental feasibility study of a patient- and family-centered rounds educational intervention that incorporated repeated direct observations during the pediatric clerkship. The study was supplemented by a retrospective historical comparison of end-of-clerkship summative evaluation scores, allowing for a single-group pre- and post-test design.

### Setting

The Core Clerkship in Pediatrics at Johns Hopkins School of Medicine is an 8-week rotation consisting of 4 weeks in a hospital setting and 4 weeks in an outpatient setting. The study was conducted at the main campus, Johns Hopkins Children’s Center, a 200-bed tertiary care pediatric hospital, and at 3 community hospitals: Ascension Saint Agnes Hospital, Sinai Hospital, and Johns Hopkins Howard County Medical Center. The study ran from October 2023 to October 2024 and included five 8-week blocks.

### Participants and intervention

Participants were medical students enrolled in the Core Clerkship in Pediatrics at a single academic medical center. All students enrolled in the clerkship during the study period were eligible, and no students were excluded on the basis of demographic characteristics. Site assignment (main campus versus community hospital) and timing of the inpatient rotation (H1 versus H2) followed standard institutional scheduling based on rotation capacity and availability; neither was randomized. Students assigned to the main campus were eligible for formative PFCR assessment, whereas students at community hospital sites attended the educational session but were not assessed with the PFCR form.

A 1-hour educational session was delivered during orientation at the start of each clerkship period, and all students attended regardless of site assignment. The session used patient cases to simulate oral presentations; students were divided into small groups, each of which role-played a case presentation as if on rounds in front of the larger group. The session was co-facilitated by a pediatric hospitalist and the manager of the hospital’s Patient and Family Advisory Council (PFAC), who role-played a parent and provided feedback from a caregiver perspective.

Students assigned to the main campus were assessed during PFCR by a pediatric faculty member, the teaching attending, using our novel assessment form. Assessments were planned for approximately once per week during each student’s 4-week hospital block. Students could receive feedback immediately after rounds and review their completed forms.

### Outcomes

The primary feasibility outcomes were (1) the overall completion rate, (2) the number of assessments completed per student, and (3) the observability of assessment domains, reflecting how well the form could be integrated into the clinical workflow of PFCR. Exploratory performance outcomes were student-level scores on the PFCR assessment form across 8 domains: (1) inviting patient and family concerns, (2) inviting nursing concerns, (3) presenting a patient summary, (4) providing an action list or plan, (5) demonstrating situational awareness, (6) ensuring synthesis by the receiver, (7) avoiding medical jargon, and (8) including the patient and family in the discussion. Secondary outcomes included end-of-clerkship summative oral presentation scores and family rapport scores.

### Data sources and measurement

The PFCR assessment form was adapted from the I-PASS SCORE program and refined through an iterative process involving medical students, pediatric faculty, and the hospital’s PFAC. The first 6 domains were rated on a 4-point ordinal scale (1=not done, 2=progressing, 3=meets expectations, and 4=exceeds expectations), and the final 2 domains were rated on a 3-point scale (1=not done, 2=a moderate amount, and 3=to a great extent). A not available (N/A) response was allowed when the target audience was absent. The form was formative and did not contribute to clerkship grades (Supplement 1).

For the pre- and post-intervention comparison, we extracted de-identified summative evaluation data from January 2021 to April 2023, allowing for a washout period before implementation, and from October 2023 to October 2024, which corresponded to the study period. Faculty attendings had submitted these scores on a 1-to-5 scale, with 1 representing “critical deficiencies” and 5 representing “mastery” (Supplement 2).

### Bias

The lack of formal rater calibration may have introduced rater variability; inter-rater reliability was not formally assessed, as acknowledged in the limitations. To promote consistency, the form included behavioral descriptors and was developed through a structured consensus process. Because students contributed different numbers of assessments, performance was summarized at the student level using medians. The assessment form was formative and ungraded, whereas summative scores contributed to clerkship grades, which may have affected rater behavior differently across the 2 instruments. Assignment of students to site and clerkship timing was determined by standard scheduling and was not randomized. Therefore, systematic differences between groups cannot be excluded and represent a potential source of selection bias. This issue is also acknowledged as a limitation of the study design.

Blinding was not implemented because of the nature of the study design, and students knew that they were being assessed. The absence of blinding may have introduced performance bias; however, because the form was formative and ungraded, we believe this effect was limited.

### Study size

The study was conducted as a feasibility evaluation, and no a priori sample size calculation was performed. All clerkship students (n=108) were eligible to participate in the workshop during the study period. The assessment-form sample comprised students rotating at the main campus during the study period (n=74). Mixed-effects models were restricted to students with at least 2 forms (n=32; 98 assessments). The pre-intervention comparison included 165 students.

### Statistical methods

Student-level median scores were calculated across all available assessment forms within each domain, excluding illegible responses and N/A designations. Group comparisons between students rotating in the first half (H1) and second half (H2) of the clerkship were performed using 2-sided Wilcoxon rank-sum tests with Holm correction for multiple comparisons. Because of ceiling effects and top-heavy score distributions, this nonparametric approach was selected as the primary analysis.

A sensitivity analysis was conducted using only each student’s first available assessment form to eliminate repeated measures. For domains that permitted N/A responses, the proportion of forms marked N/A was compared between groups using Fisher’s exact test.

To assess changes across serial assessments, linear mixed-effects models with random intercepts for students were fitted to data from students with at least 2 assessments. The slope (β) estimated the change per additional assessment, and an interaction term (assessment number×clerkship half) tested whether improvement rates differed between H1 and H2. Holm correction was applied across domains.

For summative scores, Wilcoxon rank-sum tests on student-level medians were used to compare H1 versus H2 at each site, main-campus versus community-hospital students, and pre-intervention versus study-period students at the main campus. Analyses were performed using GraphPad Prism ver. 10.0 (GraphPad Software) and R ver. 4.5.2 (R Foundation for Statistical Computing).

## Results

### Participants

During the study period, 114 students completed the Core Clerkship and received summative evaluations at our main campus or at one of 3 community hospitals. The analysis included 108 students (95%) whose summative evaluations were completed by faculty attendings.

Seventy-four students (65%) were assigned to rotate at the main campus and were eligible for formative PFCR assessment. Of these students, 45 (61%) had at least one PFCR form completed by a teaching attending, defined as a faculty observer external to the student. Among students with completed forms, 21 completed their inpatient rotation during the first half of the clerkship (H1), and 24 completed it during the second half (H2).

In total, 128 observer assessment forms were completed across the 5 clerkship blocks (Dataset 1). Of these, 98 forms were completed for the 32 students with at least 2 assessments, who were included in the mixed-effects model analyses. The number of forms per student did not differ between clerkship halves: H1 (median, 3.0; interquartile range [IQR], 1.0–4.5) vs. H2 (median, 3.0; IQR, 1.0–3.0); Wilcoxon rank-sum P=0.253.

The 108 students with faculty-completed summative evaluations comprised 73 students at the main campus (68%) and 35 students across the 3 community hospitals (32%). Two of the 73 students at the main campus were excluded from the family rapport score analysis because their evaluations were marked “not observed.” An additional 165 students from the pre-intervention period at the main campus (January 2021–April 2023) contributed to the historical comparison (Dataset 2).

### Main results

We summarized at the student level using medians across forms (Dataset 3). In both H1 and H2, student-level median scores in all domains clustered near the upper end of the scale. For domains 1–6, which were rated on a 4-point scale, group medians were ≥3.0, corresponding to “Meets expectations.” For domains 7–8, which were rated on a 3-point scale, group medians were 3.0, corresponding to “To a great extent,” the highest possible rating on that scale (Table 1).

Student performance was similar between H1 and H2 across all domains (Table 1), with no statistically significant differences between groups after Holm adjustment for multiple comparisons. The results were unchanged in a sensitivity analysis restricted to each student’s first assessment form (Dataset 4, Supplement 3).

Several domains allowed an N/A response when the target audience was not present for rounds and the student therefore could not demonstrate the relevant skill. The proportions of assessments marked N/A for inviting family concerns and inviting nursing concerns were 13% and 12%, respectively, with similar rates across halves (Fisher’s exact test, P>0.999), supporting adequate observability.

Linear mixed-effects models with student-level random intercepts were used to test whether students improved across serial assessments. Students with at least 2 assessments were included (n=32 students with 98 assessments). After Holm correction, students showed significant improvement in inviting patient and family concerns (β=0.119; 95% confidence interval [CI], 0.042–0.196; standard error [SE]=0.04; adjusted P=0.024). Unadjusted analyses also showed improvement in inviting nursing concerns (β=0.162; 95% CI, 0.036–0.289; SE=0.065; unadjusted P=0.014) and situational awareness (β=0.101; 95% CI, 0.011–0.192; SE=0.046; unadjusted P=0.030), but these associations did not remain significant after correction (adjusted P=0.098 and P=0.180, respectively). No significant change was observed in the remaining domains (Table 2).

We tested whether improvement rates differed between clerkship halves, H1 and H2, using an interaction term (assessment number×half). The interaction term was not significant for any domain (all unadjusted P>0.05), indicating that learning trajectories were similar in H1 and H2 (Table 2).

Oral presentation scores did not differ between H1 and H2 students at the main campus (both groups: median, 4.5; IQR, 4.0–5.0; P=0.775) (Table 3). Family rapport scores also did not differ (both groups: median, 4.5; IQR, 4.0–5.0; P=0.607) (Table 3). Similarly, oral presentation and family rapport scores did not differ between H1 and H2 at any community hospital site (P>0.05 for all comparisons) (Supplement 4).

Students at the main campus, where PFCR assessment forms were used during rounds, had scores similar to those of students at community hospitals, where students participated in the educational session only (main campus: median, 4.5; IQR, 4.0–5.0; community hospital: median, 5.0; IQR, 4.0–5.0 for both domains; oral presentation P=0.227; family rapport P=0.138) (Table 3).

Finally, pre-intervention oral presentation scores at the main campus (median, 4.5; IQR, 4.0–5.0) did not differ from study-period scores (median, 4.5; IQR, 4.0–5.0; P=0.542) (Table 3). Pre-intervention family rapport scores (median, 5.0; IQR, 4.0–5.0) likewise did not differ from study-period scores (median, 4.5; IQR, 4.0–5.0; P=0.143) (Table 3).

## Discussion

### Key results

This study evaluated the feasibility of implementing a simulation-based PFCR educational session paired with a structured assessment form in a pediatric clerkship. The intervention was delivered across 5 consecutive clerkship blocks. Assessment scores did not differ significantly between students rotating in the first and second halves of the clerkship, suggesting no meaningful decline in performance over time. Across serial direct observations, students showed significant improvement in inviting patient and family concerns. However, end-of-clerkship summative oral presentation and family rapport scores did not change significantly from pre-intervention values and did not differ between students who used the assessment form and those who did not.

### Interpretation

The absence of significant differences in PFCR scores between H1 and H2 students is consistent with a sustained effect of the educational session. Comparable performance across clerkship halves suggests that the session’s effect did not decay over time, which is encouraging given the challenge of skill retention in clerkship settings. Alternative explanations should also be considered, including the possibility that H2 students had accumulated additional clerkship experience before their inpatient rotation, which may have improved measured performance. However, this pattern was not detected when we analyzed only students’ first assessment forms.

The significant improvement in inviting patient and family concerns across serial assessments reflects a core interpersonal PFCR skill that may be particularly responsive to repeated practice under direct observation. Because the assessment form was formative and students could receive feedback from the observing attending after rounds, these gains may reflect the value of structured serial feedback. The fact that this was the only domain to reach significance after Holm correction may reflect ceiling effects and the limited number of assessments completed per student.

Several factors may explain the lack of change in summative end-of-clerkship scores. First, our institution’s summative anchors are not specific to PFCR objectives, limiting their sensitivity to this intervention. Second, the formative and ungraded nature of the PFCR assessment form, compared with the summative and graded nature of end-of-clerkship evaluations, may have influenced rater stringency. Third, the well-documented problem of rater variability in clinical performance assessment suggests that summative scores may reflect assessor characteristics as much as true performance.

### Comparison with previous studies

Our findings are consistent with prior work demonstrating the feasibility of workshop-based PFCR curricula for medical students. This study extends that literature by focusing on observed rather than self-reported performance. The findings also align with the 21-item Presenter Empowerment Action checklist developed by a prior group, although our form used fewer items to facilitate integration into routine clinical care [9].

### Limitations

The single-site nature of this study limits generalizability to institutions with different curricular structures or PFCR practices. The absence of formal rater calibration and the inability to link individual evaluators to specific assessments precluded adjustment for assessor-level clustering and rater effects, which may have introduced unmeasured variability. The assessment form was not completed for all eligible students (61% completion), creating possible selection bias if attendings were more likely to complete forms for higher- or lower-performing students. Although we tracked completion and observability as feasibility indicators, we did not systematically collect data on attending experience with the form or on specific barriers to completion for eligible students. Although the brief ordinal form was designed to maximize completion in the clinical setting, it may have been insufficiently granular to detect smaller changes in performance. The pre-intervention comparison relied on historical summative data, which may have differed systematically from the study-period data in ways not captured by the available variables.

### Generalizability

This study was conducted at a single academic medical center, but the core components of the intervention—a brief simulation-based session co-facilitated by a family advisory representative and paired with a structured direct-observation form—are designed to be adaptable across institutions. The form’s alignment with the widely used I-PASS framework and its use of student-appropriate behavioral anchors, distinct from resident-level expectations, may facilitate adoption at other programs. Involving a Patient and Family Advisory Council in form development and teaching also provides a transferable model for incorporating authentic family perspectives into medical education.

### Suggestions

Validation in diverse institutional contexts, including community-based programs and international settings, would strengthen the evidence base for this approach. Multi-site implementation would enable adequately powered comparisons that account for clustering within students, raters, and institutions. Rater training and calibration would strengthen future use of the form and enhance its validity evidence. Controlled study designs are needed to establish the causal benefits of workshop-based PFCR training. Future iterations should also gather structured input from faculty users about the form’s time burden and fit within clinical workflow.

### Conclusion

A structured PFCR educational session paired with a brief, student-aligned observer assessment form can be feasibly implemented within a pediatric clerkship. Observed performance met or exceeded expectations across key communication domains, although the absence of a control group precludes conclusions about the causal effect of the intervention. Serial direct observation was associated with improvement in family engagement skills. The form’s grounding in a validated framework, alignment with medical student-level expectations, and co-development with a Patient and Family Advisory Council contribute to the PFCR education literature.

## Figures and Tables

**Figure f1-jeehp-23-18:**
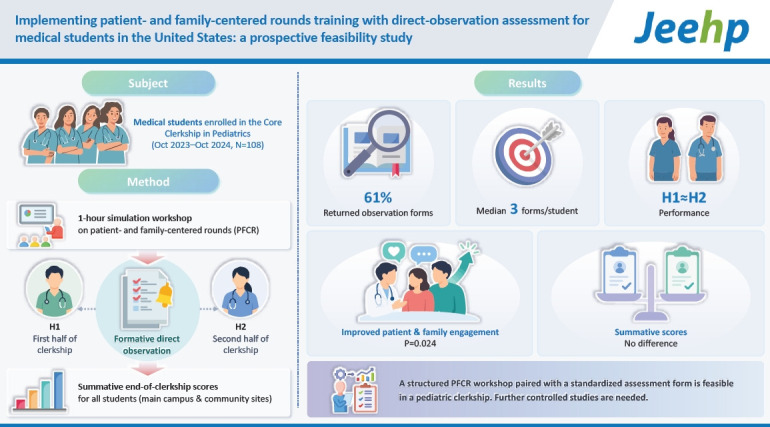


**Table 1.** Student-level performance on PFCR assessment form domains

Domain	H1		H2		Mann-Whitney U	P-value (unadjusted)	P-value (Holm correction)
	No. of students	Median (IQR)	No. of students	Median (IQR)			
Items rated on Likert-type scale 1–4							
Inviting patient and family concerns	20	3.5 (3.0–4.0)	24	3.75 (3.0–4.0)	239.5	1	1
Inviting nursing concerns	18	3.0 (3.0–4.0)	17	3.0 (3.0–4.0)	150.5	1	1
Patient summary	21	3.0 (3.0–4.0)	24	3.0 (3.0–4.0)	250.5	1	1
Action list	21	3.0 (3.0–4.0)	24	3.0 (3.0–4.0)	216	1	1
Situation awareness	21	3.0 (3.0–3.0)	23	3.0 (3.0–4.0)	193	1	1
Synthesis by receiver	21	3.0 (3.0–3.0)	22	3.0 (3.0–3.75)	227.5	1	1
Items rated on Likert-type scale 1–3							
Avoiding medical jargon	19	3.0 (2.75–3.0)	23	3.0 (2.5–3.0)	207.5	0.823	1
Including patient and family in discussion	19	3.0 (3.0–3.0)	23	3.0 (3.0–3.0)	190	0.239	1

Values are student-level median scores. Group comparisons between H1 and H2 of the clerkship period were assessed using 2-sided Wilcoxon rank-sum (Mann-Whitney U) tests. P-values were adjusted for multiple comparisons across domains (Holm method). PFCR, patient- and family-centered rounds; H1, first half of the clerkship; H2, second half of the clerkship; IQR, interquartile range.

**Table 2.** Change in student performance on PFCR domains over serial assessments and moderation by clerkship timing

Domain	Overall slope β (SE) [95% CI]	P-value (unadjusted)	P-value (Holm)	H1 slope	H2 slope	F-value	P-value (unadjusted)	P-value (Holm)
Items rated on Likert-type scale 1–4								
Inviting patient and family concerns	0.119 (0.039) [0.042 to 0.196]	0.003^*^	0.024^*^	0.112	0.120	F(1,81.5)=0.01	0.920	1
Inviting nursing concerns	0.162 (0.065) [0.036 to 0.289]	0.014^*^	0.098	0.235	0.105	F(1,77.0)=0.96	0.329	1
Patient summary	0.061 (0.042) [−0.020 to 0.143]	0.143	0.590	0.049	0.076	F(1, 105.8)=0.11	0.744	1
Action list	0.027 (0.037) [−0.045 to 0.100]	0.463	1	0.023	0.028	F(1, 95.1)=0.00	0.946	1
Situation awareness	0.101 (0.046) [0.011 to 0.192]	0.030^*^	0.180	0.126	0.080	F(1, 93.5)=0.26	0.614	1
Synthesis by receiver	0.021 (0.046) [−0.069 to 0.110]	0.652	1	0.113	−0.055	F(1, 105.1)=3.39	0.069	0.552
Items rated on Likert-type scale 1–3								
Avoiding medical jargon	−0.015 (0.030) [−0.073 to 0.043]	0.611	1	−0.041	0.018	F(1, 89.6)=0.96	0.329	1
Including patient and family in discussion	0.035 (0.022) [−0.008 to 0.078]	0.118	0.590	0.045	0.023	F(1,84.4)=0.25	0.618	1

Linear mixed-effects models with random student intercepts tested whether students improved across repeated assessments and whether the rate of change differed by clerkship half (H1 vs. H2). Overall slope β (SE) represents change in score per assessment. H1 and H2 slopes show improvement rates in each clerkship half. P-values are shown unadjusted and adjusted using Holm correction for multiple comparisons across domains. The first 6 domains were rated on a 4-point Likert-type scale; the last 2 domains were rated on a 3-point scale. H1, first half of clerkship; H2, second half of clerkship; SE, standard error.

* P<0.05 (statistically significant).

**Table 3.** Student-level performance on end-of-clerkship oral presentation and family rapport scores

Comparison	Group	Median (IQR)	Group	Median (IQR)	Mann-Whitney U	P-value (unadjusted)
H1 vs. H2 at main campus						
Oral presentation score	H1 (n=38)	4.5 (4.0–5.0)	H2 (n=35)	4.5 (4.0–5.0)	637	0.775
Family rapport score	H1 (n=36)	4.5 (4.0–5.0)	H2 (n=35)	4.5 (4.0–5.0)	590	0.607
Main campus vs. all community hospitals						
Oral presentation score	Main campus (n=73)	4.5 (4.0–5.0)	Community sites (n=35)	5.0 (4.0–5.0)	1,102	0.227
Family rapport score	Main campus (n=71)	4.5 (4.0–5.0)	Community sites (n=35)	5.0 (4.0–5.0)	1,039	0.138
Pre-intervention vs. study period at the main campus						
Oral presentation score	Pre (n=164)	4.5 (4.0–5.0)	Post (n=73)	4.5 (4.0–5.0)	5,705	0.542
Family rapport score	Pre (n=165)	5.0 (4.0–5.0)	Post (n=71)	4.5 (4.0–5.0)	5,205	0.143

Values are student-level median scores across evaluations. The Wilcoxon rank-sum test (Mann-Whitney U) was used to compare student-level medians. IQR, interquartile range; H1, first half of clerkship; H2, second half of clerkship.

## Footnotes

**Data availability**
Data files are available from https://doi.org/10.7910/DVN/G56LJP Dataset 1. Raw observational data from serial assessments during patient- and family-centered rounds (PFCR).

Dataset 2. End-of-clerkship summative evaluation scores for oral presentation and family rapport.

Dataset 3. Student-level median scores across patient- and family-centered rounds assessment domains.

Dataset 4. Cross-sectional data from medical students’ first patient- and family-centered rounds assessment.

## Supplementary materials

Supplement 1. Patient- and family-centered rounds external observer assessment form.

Supplement 2. Codebook for datasets 1–4.

Supplement 3. Sensitivity analysis restricted to each student’s first assessment form for PFCR domains.

Supplement 4. Student-level performance on end-of-clerkship oral presentation and family rapport scores at community inpatient pediatric sites.

Supplement 5. Audio recording of the abstract.
