# Supplementary material for: Implementing patient- and family-centered rounds training with direct-observation assessment for medical students in the United States: a prospective feasibility study
Source: J Educ Eval Health Prof. 2026 Jun 18;23:18. doi: 10.3352/jeehp.2026.23.18 (PMC13473693; doi:10.3352/jeehp.2026.23.18)
Supplement: Supplementary file 1 [file jeehp-23-18-suppl1.docx]

**Supplement 1. PFCR External Observer Assessment Form**

**Patient and Family-Centered Rounds (PFCR) Observation Form for Medical Students**

FORMATIVE – NOT TO BE USED IN GRADING OR EVALUATIONS

Date: _______________ Student: ___________________________ Observer: ___________________________

| **PFCR Element** | **Description** | **Behavior** |
| --- | --- | --- |
| **Inviting concerns** | Providing an opportunity for family and other team members to share concerns and questions | **Student invites patient and/or family to share concerns or questions**  ☐ Not done ☐ Progressing ☐ Meets expectations ☐ Exceeds expectations ☐ N/A (patient or family not present)  **At any point, student invites nursing to share concerns or questions**  ☐ Not done ☐ Progressing ☐ Meets expectations ☐ Exceeds expectations ☐ N/A (nursing not present) |
| Comments: |  |  |
| **Patient summary** | Pertinent history, exam, data, assessment (may be in SOAP format) | **Student presents updates, relevant data, and overall assessment**  ☐ Not done ☐ Progressing ☐ Meets expectations ☐ Exceeds expectations |
| Comments: |  |  |
| **Action list** | Appropriate plan with specific action items for the next 24 hours | **Student outlines concrete plan based on clinical assessment**  ☐ Not done ☐ Progressing ☐ Meets expectations ☐ Exceeds expectations |
| Comments: |  |  |
| **Situation awareness** | Things to look out for during admission and anticipating what might happen | **While presenting plan, student outlines 1-2 possible scenarios and what interventions or management steps may be indicated**  ☐ Not done ☐ Progressing ☐ Meets expectations ☐ Exceeds expectations |
| Comments: |  |  |
| **Synthesis by receiver** | Confirming shared understanding among family and team members | **Student synthesizes presentation OR invites family or resident to share their understanding of the care plan**  ☐ Not done ☐ Progressing ☐ Meets expectations ☐ Exceeds expectations |
| Comments: |  |  |

**To what extent did the student avoid using medical jargon?**

☐ Not at all ☐ A moderate amount ☐ To a great extent ☐ Patient/family not present

**To what extent did the student include the patient and family in the discussion?**

☐ Not at all ☐ A moderate amount ☐ To a great extent ☐ Patient/family not present
