# Supplementary material for: Implementing patient- and family-centered rounds training with direct-observation assessment for medical students in the United States: a prospective feasibility study
Source: J Educ Eval Health Prof. 2026 Jun 18;23:18. doi: 10.3352/jeehp.2026.23.18 (PMC13473693; doi:10.3352/jeehp.2026.23.18)
Supplement: Supplementary file 2 [file jeehp-23-18-suppl2.docx]

**Codebook**

Four datasets are described below. Assessment domain scores reflect performance on the PFCR external observer assessment form, developed and used formatively during the prospective study period (October 2023–October 2024).

# Dataset 1. Raw observational data of serial assessments on patient- and family-centered rounds (PFCR) (“dataset_1_observed.csv”)

Contains one row per assessment form completed. Students with multiple forms appear in multiple rows. Used for mixed-effects models examining change across serial assessments.

| **Variable** | **Description** | **Values / Notes** |
| --- | --- | --- |
| student_id | De-identified numeric student identifier | Integer |
| half | Clerkship half in which the student completed the hospital-based rotation | H1 = First half of clerkship block; H2 = Second half |
| assessment_number | Sequential number of the assessment form for that student | Integer starting at 1. Increments with each additional form completed for the same student |
| eval_id | De-identified unique identifier for the individual assessment form | Integer. Unique across all forms in the dataset |
| family_concerns | Student invited patient and family to share concerns during rounds | 4-point scale: 1 = Not done; 2 = Progressing; 3 = Meets expectations; 4 = Exceeds expectations. Blank = N/A recorded in family_NA column |
| nursing_concerns | Student invited nursing staff to share concerns during rounds | 4-point scale (same as above). Blank = N/A recorded in nursing_NA column |
| patient_summary | Student presented a pertinent patient summary | 4-point scale (same as above) |
| action_list | Student provided a clear action list or plan | 4-point scale (same as above) |
| situation_awareness | Student demonstrated situational awareness during rounds | 4-point scale (same as above) |
| synthesis_by_receiver | Student ensured synthesis by the receiver | 4-point scale (same as above) |
| family_NA | Indicates that the family was not present and student had no opportunity to demonstrate family-related skills | N/A = not applicable; blank = domain was scored |
| nursing_NA | Indicates that nursing staff were not present and student had no opportunity to demonstrate nursing engagement | N/A = not applicable; blank = domain was scored |
| medical_jargon | Student avoided medical jargon when speaking with family | 3-point scale: 1 = Not done; 2 = A moderate amount; 3 = To a great extent. N/A = not applicable |
| family_inclusion | Student included the patient and family in the discussion | 3-point scale (same as above). N/A = not applicable |

# Dataset 2. Cross-sectional data of medical students' first patient- and family-centered rounds assessment (“dataset_2_first_form.csv”)

Contains one row per student, restricted to each student’s first available assessment form. Identical column structure to Dataset 1 (see above). Used as a sensitivity analysis.

# Dataset 3. Student-level median scores across patient- and family-centered rounds assessment domains (“dataset_3_student_median.csv”)

Contains one row per student. Domain scores represent the median across all available assessment forms for that student. Used as the primary dataset for H1 vs. H2 cross-sectional comparisons using Wilcoxon rank-sum tests.

| **Variable** | **Description** | **Values / Notes** |
| --- | --- | --- |
| student_id | De-identified numeric student identifier | Integer. Consistent with datasets 1 and 2 |
| half | Clerkship half assignment | H1 = First half; H2 = Second half |
| family_concerns | Student-level median score for inviting patient and family concerns, calculated across all available assessment forms | Numeric (1–4). Blank if all forms for this student were marked N/A |
| propNA_family_concerns | Proportion of assessment forms for this student marked N/A for family_concerns | Numeric (0–1). 1 = all forms marked N/A |
| nursing_concerns | Student-level median score for inviting nursing concerns | Numeric (1–4). Blank if all forms marked N/A |
| propNA_nursing_concerns | Proportion of assessment forms marked N/A for nursing_concerns | Numeric (0–1) |
| patient_summary | Student-level median score for presenting a pertinent patient summary | Numeric (1–4) |
| action_list | Student-level median score for providing a clear action list or plan | Numeric (1–4) |
| situation_awareness | Student-level median score for demonstrating situational awareness | Numeric (1–4) |
| synthesis_by_receiver | Student-level median score for ensuring synthesis by the receiver | Numeric (1–4) |
| medical_jargon | Student-level median score for avoiding medical jargon | Numeric (1–3). Blank if all forms marked N/A |
| family_inclusion | Student-level median score for including the patient and family in discussion | Numeric (1–3). Blank if all forms marked N/A |
| total_forms | Total number of assessment forms completed for this student | Integer ≥1 |

# Dataset 4. End-of-clerkship summative evaluation scores (oral presentation and family rapport) (“dataset_4_summative.csv”)

Contains one row per student. Includes students from both the pre-intervention period and the prospective study period. Used for comparisons of summative oral presentation and family rapport scores across clerkship halves, sites, and study periods.

| **Variable** | **Description** | **Values / Notes** |
| --- | --- | --- |
| subject | De-identified student identifier for summative dataset | String (e.g., Subject_001). Not linked to student_id in datasets 1–3 to protect anonymity |
| eval_status | Indicates whether the evaluation was from the pre-intervention or study period | pre = pre-intervention period; study = prospective study period |
| half | Clerkship half in which the student completed the hospital-based rotation | H1 = First half; H2 = Second half. Pre-intervention students assigned based on clerkship block timing |
| location | Hospital site where the student completed the hospital-based portion of the clerkship | Main Campus = 200-bed tertiary care pediatric hospital; Community Hospital 1, 2, or 3 = affiliated community sites |
| median_oral_presentation | Student-level median end-of-clerkship oral presentation score | Numeric (1–5): 1 = Critical deficiencies; 5 = Mastery. Calculated from faculty attending evaluations; “not observed” responses excluded |
| median_family_rapport | Student-level median end-of-clerkship family rapport score | Numeric (1–5), same scale as oral presentation score |
| n_observations | Number of faculty evaluations contributing to the student’s median scores | Integer ≥1. “Not observed” responses excluded from count |
