# Supplementary material for: Implementing patient- and family-centered rounds training with direct-observation assessment for medical students in the United States: a prospective feasibility study
Source: J Educ Eval Health Prof. 2026 Jun 18;23:18. doi: 10.3352/jeehp.2026.23.18 (PMC13473693; doi:10.3352/jeehp.2026.23.18)
Supplement: Supplementary file 3 [file jeehp-23-18-suppl3.docx]

**Supplement 3.** Sensitivity analysis restricted to each student’s first assessment form for PFCR domains

| Domain | H1 | | H2 | | Mann-Whitney U | P-value (unadjusted) |
| --- | --- | --- | --- | --- | --- | --- |
|  | No. of students | Median (IQR) | No. of students | Median (IQR) |  |  |
| Inviting patient and family concerns | 17 | 3.0 (3.0–4.0) | 21 | 3.0 (3.0–4.0) | 172 | 0.831 |
| Inviting nursing concerns | 13 | 3.0 (1.5–4.0) | 16 | 3.0 (3.0–4.0) | 85 | 0.412 |
| Patient summary | 21 | 3.0 (3.0–4.0) | 24 | 3.5 (2.25–4.0) | 225 | 0.530 |
| Action list | 20 | 3.0 (3.0–4.0) | 24 | 3.0 (3.0–4.0) | 240 | 1 |
| Situation awareness | 19 | 3.0 (3.0–4.0) | 23 | 3.0 (2.0–4.0) | 203 | 0.691 |
| Synthesis by receiver | 20 | 3.0 (3.0–3.75) | 23 | 3.0 (3.0–4.0) | 200.5 | 0.440 |
| Avoiding medical jargon (scale 1–3) | 16 | 3.0 (3.0–3.0) | 22 | 3.0 (2.75–3.0) | 158 | 0.675 |
| Including patient and family in discussion (scale 1–3) | 15 | 3.0 (3.0–3.0) | 21 | 3.0 (3.0–3.0) | 203 | 0.253 |

To eliminate repeated measures, analyses were repeated using only the first available assessment form for each student. Group differences were assessed using 2-sided Wilcoxon rank-sum tests; all P-values were 1 after Holm correction for multiple comparisons.

PFCR, patient- and family-centered rounds; H1, first half of clerkship; H2, second half of clerkship; IQR, interquartile range.
