# Supplementary material for: Implementing patient- and family-centered rounds training with direct-observation assessment for medical students in the United States: a prospective feasibility study
Source: J Educ Eval Health Prof. 2026 Jun 18;23:18. doi: 10.3352/jeehp.2026.23.18 (PMC13473693; doi:10.3352/jeehp.2026.23.18)
Supplement: Supplementary file 4 [file jeehp-23-18-suppl4.docx]

**Supplement 4.** Student-level performance on end-of-clerkship oral presentation and family rapport scores at community inpatient pediatric sites

| Site | H1 | | H2 | | Mann-Whitney U | P-value (unadjusted) |
| --- | --- | --- | --- | --- | --- | --- |
|  | No. of students | Median (IQR) | No. of students | Median (IQR) |  |  |
| Oral presentation score |  |  |  |  |  |  |
| Community hospital A | 11 | 5.0 (4.5–5.0) | 8 | 5.0 (4.6–5.0) | 41 | 0.722 |
| Community hospital B | 3 | 5.0 (4.0–5.0) | 3 | 5.0 (3.0–5.0) | 4 | 1 |
| Community hospital C | 5 | 4.0 (4.0–4.0) | 5 | 4.0 (3.0–4.0) | 7.5 | 0.444 |
| Family rapport score |  |  |  |  |  |  |
| Community hospital A | 11 | 4.5 (4.0–5.0) | 8 | 5.0 (4.3–5.0) | 32 | 0.352 |
| Community hospital B | 3 | 5.0 (5.0–5.0) | 3 | 5.0 (4.0–5.0) | 3 | 1 |
| Community hospital C | 5 | 5.0 (4.0–5.0) | 5 | 4.0 (3.0–4.0) | 8 | 0.444 |

Within each analysis subset, scores were collapsed to the student level by using each student’s median score across evaluations. Two-group comparisons used the Wilcoxon rank-sum test (Mann-Whitney U) on student-level medians; all P-values were 1 after Holm adjustment for multiple comparisons.

H1, first half of clerkship; H2, second half of clerkship; IQR, interquartile range.
